# Research Progress of Steels for Nuclear Reactor Pressure Vessels

**DOI:** 10.3390/ma15248761

**Published:** 2022-12-08

**Authors:** Linjun Zhou, Jie Dai, Yang Li, Xin Dai, Changsheng Xie, Linze Li, Liansheng Chen

**Affiliations:** 1 College of Metallurgy and Energy, North China University of Science and Technology, Tangshan 063210, China; 2 College of Marxism Institute, Hebei College of Science and Technology, Shijiazhuang 063210, China; 3 College of Electronic and Optical Engineering and Flexible Electronics (Future Technology), Nanjing University of Posts and Telecommunications, Nanjing 210023, China; 4 Technology Center, Zenith Steel Group (Nantong) Co., Ltd., Changzhou 226156, China

**Keywords:** nuclear reactor pressure vessels, microstructure evolution, mechanical properties, irradiation, corrosion, thermal aging, fatigue properties

## Abstract

The nuclear reactor pressure vessel is an important component of a nuclear power plant. It has been used in harsh environments such as high temperature, high pressure, neutron irradiation, thermal aging, corrosion and fatigue for a long time, which puts forward higher standards for the performance requirements for nuclear pressure vessel steel. Based on the characteristics of large size and wall thickness of the nuclear pressure vessel, combined with its performance requirements, this work studies the problems of forging technology, mechanical properties, irradiation damage, corrosion failure, thermal aging behavior and fatigue properties, and summarizes the research progress of nuclear pressure vessel materials. The influencing factors of microstructures evolution and mechanism of mechanical properties change of nuclear pressure vessel steel are analyzed in this work. The mechanical properties before and after irradiation are compared, and the influence mechanisms of irradiation hardening and embrittlement are also summarized. Although the stainless steel will be surfacing on the inner wall of nuclear pressure vessel to prevent corrosion, long-term operation may cause aging or deterioration of stainless steel, resulting in corrosion caused by the contact between the primary circuit water environment and the nuclear pressure vessel steel. Therefore, the corrosion behavior of nuclear pressure vessels materials is also summarized in detail. Meanwhile, the evolution mechanism of the microstructure of nuclear pressure vessel materials under thermal aging conditions is analyzed, and the mechanisms affecting the mechanical properties are also described. In addition, the influence mechanisms of internal and external factors on the fatigue properties, fatigue crack initiation and fatigue crack propagation of nuclear pressure vessel steel are analyzed in detail from different perspectives. Finally, the development direction and further research contents of nuclear pressure vessel materials are prospected in order to improve the service life and ensure safe service in harsh environment.

## 1. Background

In recent years, the global energy crisis has swept the world. Fossil energy, such as coal, oil and natural gas, is being consumed at a visible rate, resulting in energy shortages. Currently, the world is dominated by coal power generation. However, the coal power generation not only causes environmental pollution but also leads to energy exhaustion, which will cause the global power shortage and price rise [1,2]. Nuclear energy is a kind of clean, efficient, economical and safe renewable energy [3]. Nuclear energy is one of the effective ways to solve the energy crisis. However, there has been a global anxiety about the use of nuclear power since the Fukushima accident. Therefore, research on the safety of nuclear power plants and their components is the key to the global nuclear power industry.

### 1.1. Development of Steel for Nuclear Pressure Vessels

Nuclear reactor pressure vessel (RPV) is an important component of nuclear power plant and cannot be replaced during the entire life cycle. Therefore, the steel used for RPV was generally improved on the basis of the previous generation of RPV materials. The initial material of RPV was C–Mn steel used for the boiler. The plate of SA212B, and the forgings of SA105 and SA182, were selected as the steels for first-generation RPV due to their good welding performance and high strength. However, the impact toughness and high temperature performance of C–Mn steel are poor, and the hardenability was insufficient, so the first generation of nuclear pressure vessel steel has been replaced.

The first generation RPV material was replaced by Mn–Mo-series low-alloy high-strength steel SA302B plate in order to improve the strength and toughness, which was called the second generation of RPV steel. Subsequently, the modified SA533B was made by adding 0.4–1% Ni element on the basis of SA302B, which had good strength, hardness and toughness, so it was widely used in nuclear reactor pressure vessels [4,5]. RPV materials would be damaged by strong neutron radiation during the service. However, there were more longitudinal and circumferential welds in the plates of the first and second generation RPV steels, and the position of the welds was the weak link of radiation resistance. Therefore, in order to increase the safety and reliability of nuclear pressure vessels during the service, forging materials were gradually used to decrease the welding areas. Then, the SA508Gr.2 steel was improved on the basis of SA105 and SA182 forgings by adding Ni and Ni–Mo elements. However, SA508Gr.2 steel was gradually eliminated due to insufficient hardenability, poor toughness, and reheat cracks under the surfacing layer [6].

The possibility of reheat cracks in SA508Gr.2 steel was reduced by decreasing the contents of C, Cr and Mo. Meanwhile, the Mn was added to improve the strength of RPV materials. Therefore, SA508Gr steel was improved as the third-generation nuclear reactor pressure vessel material under this background [7]. At present, SA508Gr.3 steel is the preferred material for RPVs, which decreases the area of weld joints and greatly improves the radiation resistance as well as the overall safety of nuclear power plants. Meanwhile, the widely used third-generation RPV materials also include 20MnMoNi55 steel in Germany [8,9,10], 16MnD5 steel in France [11,12], 15X2HM steel in Russia [13,14] and SA508Gr.3 steel in China [15], and so on.

With the improvement of safety performance and increase of service life of nuclear power plants, RPV materials are developing towards the direction of “large-scale integrated design” and “high safety and longevity operation”, which requires the steel used in RPV to have better hardenability and higher strength and toughness [16,17,18]. When using SA508Gr.3 steel, it was difficult to ensure the uniformity of microstructure and the stability of properties on the extra-thick section due to the insufficient hardenability [19]. Therefore, SA508Gr.4N steel was used as the new generation nuclear pressure vessel material by increasing Ni and Cr elements and decreasing Mn element based on SA508Gr.3 steel [20]. The reduction of Mn content in SA508Gr.4N steel would decrease segregation and make the interior of the material pure, and the increase of Ni element could improve the hardenability [21]. Moreover, increasing Cr content could promote the precipitation of precipitates and refine carbides [22]. SA508Gr.4N steel was considered as candidate structural material for the new generation RPV because these had higher hardenability, better mechanical and irradiation properties compared with SA508Gr.3 steel [19,23,24,25]. The main chemical composition content of different nuclear pressure vessel materials and A508 series steel are shown in Table 1 and Table 2, respectively.

### 1.2. Service Environment of Steel for Nuclear Pressure Vessel

The safety of nuclear power plant depends on the reliability of nuclear island equipment, especially the nuclear vessel equipment that directly or indirectly contact with radioactive media, such as nuclear reactor pressure vessels, steam generators, pressurizers, etc., and the RPV also plays a role in maintaining the operating pressure balance in the reactor. RPV materials were constantly exposed to high temperature and high pressure during the service. They determined the safety and service life of nuclear power plant to a great extent [27,28,29,30]. The nuclear reactor pressure vessel contained the reactor core to prevent the leakage of radioactive substances. Therefore, the radiation damage would accompany the whole service life of RPV. Table 3 showed the neutron fluence rate and the neutron fluence in the whole service cycle of common reactor types. It could be seen from the Table 3 that the nuclear reactor was seriously affected by neutron irradiation during the service cycle, which would cause deterioration of the performance of nuclear pressure vessel materials.

In addition, the RPV nozzle was also connected with the primary circuit main pipeline cooling system. The RPV materials were not resistant to corrosion, so a layer of austenitic stainless steel or nickel-based alloy corrosion-resistant lining would be overlaid on its inner wall to prevent corrosion. The primary cooling system was isolated from the outside world and oxygen concentration was very low, which would not cause corrosion damage to the nuclear pressure vessel system during the normal service conditions. However, the damage to the corrosion-resistant surfacing layer, stress corrosion cracking of alloy pipe penetrations at the bottom and upper closure of nuclear pressure vessels and potential leakage sources (flanges, bolts, sealing rings, valves) would lead to corrosion behavior and decrease the service life of materials during the long-term service of RPV. Therefore, corrosion was also one of the service environments of the RPV.

**Figure 1:**
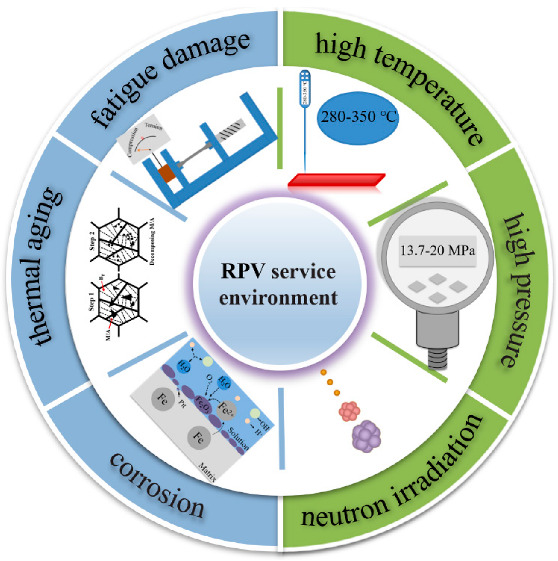
The service environment of nuclear pressure vessel [27,29,31,32,38,42].

The service life of nuclear power plants had been increased from the original design of 40 years to 60 years with the rapid development of nuclear power technology, and it would be extended to 80 years in the future [32,33,34]. The long-term service of nuclear pressure vessel at high temperature would lead to the thermal aging behavior of the RPV materials, which would affect its microstructures and properties [35,36,37,38]. So, the service environment of RPV also included thermal aging. In addition, the RPV would be affected by fatigue damage during the service. The frequent temperature fluctuations, the start-up and shut-down process, the process of emergency shutdown and unloading would cause the RPV subjected to the influence of cyclic thermal stress, which would cause continuous fatigue damage behavior of the structural components during their lifespan [9,39,40,41]. Therefore, the fatigue damage of RPV materials was an important failure mode during the service. In summary, the service environment of RPV included high temperature, high pressure, neutron irradiation, corrosion, thermal aging and fatigue damage, as shown in Figure 1. During the long-term operation of nuclear pressure vessels, it not only the single service environment damaged the matrix of materials, but also the synergistic damaged of various damage mechanisms, which might cause the material to fail to meet the design standards and be scrapped in advance in the later stage of service. In this review, the effect of service environments on RPV materials are discussed one-by-one.

## 2. Hot Deformation Behavior of Nuclear Pressure Vessel

RPV materials were subjected to different stages before application, such as smelting, ingot casting, forging, preheat-treatment, rough machining, quenching and tempering heat treatment, post-weld heat treatment, and delivery. Once the forging parameters were not well controlled during the forging, it was easy to cause mixed-crystal microstructures and other defects in the RPV materials, which would seriously influence the safe service performance [16,17,43]. Therefore, it was essential to study the hot deformation behavior for RPV materials. Dong et al. [44] established the dynamic recrystallization constitutive equations of SA508Gr.3 steel by hot compression test and verified its accuracy through combining numerical simulation with experiments, which meant that the numerical simulation platform could effectively guide the forging of industrial SA508Gr.3 steel. The deformation of the nuclear pressure vessel materials was not uniform due to the large size of the forgings during the industrial forging, which would lead to different grain sizes of the forgings. Sui et al. [45] studied the change of grain size of SA508Gr.3 steel under inhomogeneous hot deformation conditions, as well as investigating the effects of temperature and strain on the microstructure evolution during the inhomogeneous plastic deformation by numerical simulation, and found that the simulation results consistent with the experimental results.

**Figure 2:**
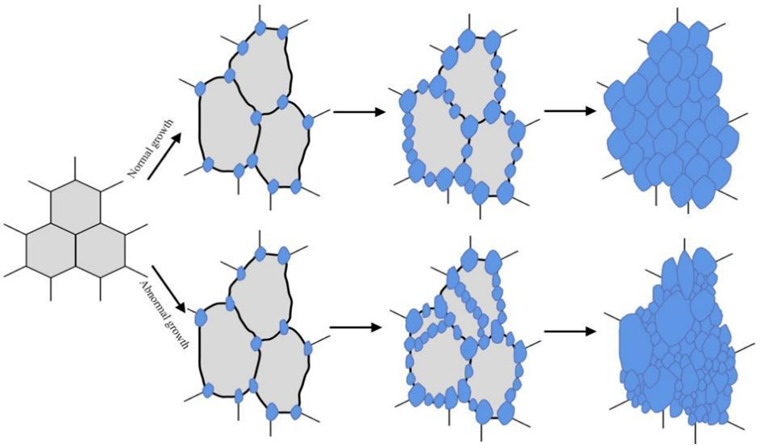
Schematic illustration of the DRX microstructures evolution of the RPV materials with the increase of strain [17,49].

Dai et al. [17] studied the dynamic recrystallization behaviors of SA508Gr.4N steel at different conditions. They found that the SA508Gr.4N steel could not undergo complete dynamic recrystallization under the conditions of low temperature and large strain rate, and the prior austenite grains were elongated these conditions. The complete dynamic recrystallization behavior occurred with the increase of deformation temperature and the decrease of strain rate, and the prior austenite grains were refined. However, the microstructure of the material also grew abnormally when the temperature increased to 1250 °C and the strain rate decreased to 0.001 s^−1^. The dynamic recrystallization process and abnormal grain growth process were shown in Figure 2. This was mainly due to the fact that the high temperature and low strain rate were conducive to atomic diffusion and promoted grain growth. Finally, they concluded that the SA508Gr.4N steel would obtain uniform microstructure when the forging in the temperature ranged from 1050 to 1175 °C and the strain rates 0.01–0.1 s^−1^.

The materials would be in a high temperature state after hot deformation, which would lead to the materials to undergo static recovery, static recrystallization and grain growth behaviors. Qiao et al. [46] studied the effect of pre-deformation and initial grain size on metadynamic dynamic recrystallization behavior of SA508Gr.4N steel, and found that the pre-deformation and initial grain size had little affected on the metadynamic dynamic recrystallization grain size. Subsequently, they [47] investigated the static recrystallization behavior of SA508Gr.4N steel by two-pass hot compression test at different conditions. The volume fraction of static recrystallization augmented with the increase of the first-pass strain under the same conditions. The increase of strain was conducive to the increase of dislocation density, thus improving the deformation stored energy of static recrystallization and promoting the nucleation rate of recrystallization. In addition, the static recrystallization volume fraction of SA508Gr.4N steel increased with the increase of hot compression temperature and the decrease of initial austenite grain size. Dong et al. [48] investigated the effect of first-pass strain and initial austenite grain size on static recrystallization behaviors of SA508Gr.3 steel, the results were agreement with Qiao et al. [47].

## 3. Mechanical Properties of Steels for Nuclear Pressure Vessel

The ASTM standard [24] specifies that nuclear pressure vessels must meet certain mechanical properties requirements after forging, as shown in Table 4. Therefore, it is very important to understand the factors influencing the mechanical properties of nuclear pressure vessel materials to improve their mechanical properties. The mechanical properties of RPV materials are affected by many factors, such as alloying elements, heat treatment parameters, carbides, grain boundaries, segregation as well as hydrogen charging environment, etc.

### 3.1. Effect of Alloying Elements on Mechanical Properties

The change of alloying element content is directly reflected in the change of mechanical properties, but the essential factor is the change of microstructure. For example, carbon content increases the strength of nuclear pressure vessel materials, Ni can improve the hardenability of nuclear pressure vessel materials, and Cr element can refine carbides size. Kim et al. [23] found that the higher the carbon content of SA508Gr.3 steel, the finer the cementite in the microstructure, which resulted in better fracture toughness. The Ni, Cr and Mo elements had great influence on the yield strength (YS), ultimate tensile strength (UTS) and fracture toughness of SA508Gr.4N steel [22,50,51,52]. The addition of Ni not only improved the ultimate tensile strength of SA508Gr.4N steel, but also improved its toughness, mainly because the increase of Ni content could refine the effective grain size and martensite block as well as increase the volume fraction of martensite after quenching. The increase of Mo content could improve the ultimate tensile strength of SA508Gr.4N steel, which was attributed to solid solution strengthening, precipitation strengthening and fine M_2_C carbides. The increase of Cr element had no effect on the ultimate tensile strength of SA508Gr.4N steel, but the toughness could be improved by refinement the size of carbides. Table 5 showed the test steel with different chemical compositions, and Table 6 showed the test results of mechanical properties.

### 3.2. Effect of Heat Treatment on Mechanical Properties

Heat treatment process can change the microstructure of materials, such as refining carbides, increasing high-angle grain boundaries (HAGBs), and then change the mechanical properties of materials. Ahn et al. [53] added the intercritical heat treatment process before the quenching and tempering heat treatment, and studied the influence of intercritical heat treatment on the toughness of SA508Gr.3 steel. They found that the addition of intercritical heat treatment could improve the toughness of the material from two aspects: Firstly, the microstructure was a mixed structure of tempered martensite and bainite after intercritical heat treatment, and the volume fraction of sub-grain boundaries increased, which could hinder the crack propagation; Second, the coarse and long rod-shaped carbides were spheroidized after intercritical heat treatment, and the inter-carbides distance increased, which could decrease the crack initiation position during the deformation.

The RPV materials have the characteristic of large size and large wall thickness, which will cause the wall thickness effect during the quenching and tempering heat treatment. As a result, the cooling rate gradually decreased along the surface of the nuclear pressure vessel material toward the center. Martensite structure was easy to form on the surface due to the high cooling rate, mixed structure of martensite and bainite was easy to form in the transition layer, and bainite or ferrite structure was easy to form in the center due to the low cooling rate. On the other hand, the larger the cooling rate, the smaller the grain size and martensite block size of the material, which would lead to the materials with more volume fraction of HAGBs, and then improved the mechanical properties.

Yang et al. [19] obtained the critical cooling rate of SA508Gr.4N steel as 35 °C/min by CCT curve. Martensite was obtained when the cooling rate was greater than the critical cooling rate, and otherwise granular bainite was obtained. Then, they calculated by EBSD technology that SA508Gr.4N steel with martensite microstructure had better mechanical properties due to more HAGBs. Lee et al. [54] studied the effects of 3 °C/min, 28.2 °C/min and 960 °C/min on the mechanical properties of SA508Gr.4N steel, respectively. They found that the volume fraction of martensite increased with the increase of cooling rate, as shown in Table 7. The yield strength and toughness were increased with the increase of cooling rate, which was mainly attributed to the fact that the larger the cooling rate, the smaller the grain size and carbides. However, the bainite structure had higher work hardening ability for martensite with 99% volume fraction, so the latter had higher tensile strength. Yan et al. [55] studied the effect of different cooling rates on the mechanical properties of SA508Gr.3 steel. The hardenability of SA508Gr.3 was poorer than that of SA508Gr.4N steel. The microstructure of the mixture of bainite and ferrite changed into bainite and martensite successively with the increase of cooling rate (0.02 K/s, 0.65 K/s and 20 K/s). Meanwhile, the impact toughness of the SA508Gr.3 steel increased significantly with the increase of cooling rate, which was also attributed to the increase of volume frac-tion of HAGBs, and the results are consistent with the investigation of Yang et al. and Yan et al. 

The core structure of nuclear pressure vessels steel is generally granular bainite due to the large wall thickness of nuclear pressure vessels. Dai et al. [56] and Li et al. [57,58] investigated the influence of tempering parameters on the mechanical properties of the RPV materials with granular bainite microstructure. Granular bainite was composed of M/A islands and bainitic ferrite (B_F_) matrix. The M/A islands would decompose and be divided into five stages during the tempering: Firstly, the M/A islands decomposed into carbides and bainite ferrite matrix; Secondly, the carbides spheroidize and growth with the increase of tempering time; Thirdly, the phenomenon of merging of the lath boundaries would be occurring; Fourthly, carbides growth at grain boundaries; Finally, the new M/A islands were formed when the tempering temperature exceeded the AC_1_. The carbon content in M/A islands was much higher than that in B_F_ matrix, and M/A islands belong to strengthening phase compared with the B_F_ matrix. The tensile strength of the nuclear pressure vessel material decreased, and the toughness increased with the strengthening phase decomposed into the fine carbides and bainite ferrite matrix. However, the impact toughness of the material decreased when the fine carbides were coarsened. In addition, Xie et al. [15] added low temperature pre-tempering process at 400 °C after intercritical heat treatment tempering, and studied the effect of pre-tempering on toughness of SA508Gr.3 steel. They found that the addition of pre-tempering could promote the decomposition of M/A islands in granular bainite into fine carbides, which would decrease the nucleation position of micro-cracks and improve the impact toughness of materials. In addition, the addition of the pre-tempering process could refine the size of the block and increase the HAGBs fraction, which could effectively prevent the propagation of micro-cracks. Therefore, the toughness of SA508Gr.3 steel was improved by two factors together.

### 3.3. Effect of Carbides Size on Mechanical Properties

The size of carbides had a significant effect on the impact toughness of SA508Gr.3 steel, Lee et al. [59] found that the interface between coarse carbides and matrix was prone to initiate voids during the deformation, while there were almost no voids around fine or fibrous carbides. In other words, micro-cracks were more likely to initiate around carbides of exceeding the critical size and to propagate into the matrix. Wu et al. [60] compared the tensile strength and impact toughness of SA508Gr.3 steel and SA508Gr.4N steel through the combination of experiment and finite element method. They found that the latter had better mechanical properties attribute to smaller grains, finer and more homogenous carbides.

### 3.4. Effect of Hydrogen Charging Conditions on Mechanical Properties

Where the nuclear pressure vessel material was connected to the primary high-temperature water environment. The high-temperature water would decompose H during the operation of the nuclear power plant, which might lead to the RPV materials to absorb H and thus affect its mechanical properties. Liu et al. [61,62] investigated the content of H on SA508Gr.3 steel mechanicals properties, they found that the tensile strength and yield strength of SA508Gr.3 steel changed little, but the elongation and impact toughness decreased significantly with the increase of H, and the impact fracture type changed from micro-void coalescence fracture to brittle and dimple mixed fracture. The carbides could capture H element and form a Cottrell atmosphere around it, which would reduce the bonding force between the carbides and the matrix, and then lead to the crack initiation and increase the crack propagation rate during the deformation. However, Singha et al. [63] compared the tensile properties of hydrogen charged and un-charged SA508Gr.3 steels, and found that the tensile strength of the hydrogen-charged specimen decreased, but the yield strength increased. The hydrogen trapped in the Luders bands during the deformation contributed to the dislocation movement, which would cause local strain in the Luders bands, resulted in the formation of micro-cracks. In addition, the tensile fracture of un-charged hydrogen specimen was ductile fracture, and its surface was covered by dimples, while the fracture of hydrogen charged specimen was brittle fracture and accompanied by a small number of dimples.

Wu et al. [64] studied the effect of temperature and strain rate on the tensile properties of hydrogen charged SA508Gr.3 steel, and found that the hydrogen effect was dependent on the strain rate. The elongation of the material decreased with the decrease of the strain rate at low temperature, while the elongation of the material decreased significantly with the increase of the strain rate at high temperature, which was related to the strain rate and dislocation movement. The interface between inclusions and matrix as well as carbides and matrix were traps for arresting H atoms. The dissolved hydrogen was mainly transferred to the trap through dislocation movement during the deformation, and the micro-void was formed when the local H concentration reached the critical value. At higher strain rates, the dissolved H could not move rapidly to the interface with the dislocation, so the quasi-cleavage fracture occurred at the trapping location. The dislocation motion promoted the transfer of dissolved H to the trapping sites with the decrease of strain rate, the cleavage plane appeared near the interface, and the elongation decreased.

The above research results showed that the increase of Ni and Mo content could improve the hardenability of the materials, which was beneficial to obtain martensite microstructure of nuclear pressure vessel steel. In addition, the increase of Ni content could also refine the grain size and martensite block, which would lead to higher HAGBs. The increase of Mo and Cr content could refine the carbides and made it uniformly distributed on the matrix. HAGBs and fine carbides could effectively prevent crack propagation and improve the mechanical properties of materials.

Heat treatment parameters included cooling rate, heating temperature and holding time. The cooling rate of the heat treatment gradually decreased from the surface to the center with the increase of the wall thickness of the RPV materials, the grain size and the martensite block size increased, the volume fraction of the HAGBs decreased, and the mechanical properties of the materials deteriorated. In addition, the granular bainite would decompose into ferrite and fine carbides during the quenching and tempering heat treatment, which would decrease the tensile strength of the material, but was beneficial to improve its impact toughness.

There was no consistent conclusion on the influence of H content on the tensile strength of nuclear pressure vessel materials, but it could be determined that the increase of H content in materials would significantly deteriorate the impact toughness, and the fracture type would change from ductile fracture to brittle fracture. This was mainly attributed to the fact that the carbides could capture H and form Cottrell atmosphere around it, which would cause the reduction of the bonding force between carbides and matrix, and then formed cracks.

## 4. Irradiation Properties of Steels for Nuclear Pressure Vessel

The irradiation damage process of materials can be defined as the process that the incident particles transfer energy to the target, which leads to the redistribution of target atoms in the target. DPA (displacements per atom) is the number of times the atoms in a material leave the equilibrium position, and it is the basic unit of irradiation damage of a material [65,66,67]. The movement of point defects and defect clusters will occur in the process of irradiation damage [68]. The irradiation effect is the change of physical and mechanical properties caused by the movement of these defects [69]. The reactor core is wrapped inside the RPV, and its material is exposed to neutron irradiation for a long time. The microstructures will change when the nuclear pressure vessel materials are subjected to irradiation damage for a long time, such as matrix damage and impurity element segregation at grain boundaries, etc. The change of microstructures after irradiation will lead to the change of properties, such as irradiation hardness, mechanical properties and irradiation embrittlement.

To investigate the effect of irradiation on hardness, Bai et al. [25] irradiated SA508Gr.4N steel with 3.5 MeV Fe^2+^ at 290 °C, and found that the hardness of SA508Gr.4N steel increased with the increase of irradiation doses (1, 2, 3, 10, 20, 30 dpa). Shimodaira et al. [70] studied the effect mechanism of different irradiation doses on irradiation hardening of SA508Gr.3 steel. It was found that the irradiation hardening of SA508Gr.3 steel mainly came from three aspects: First, Ni, Mn and Si solute clusters would be formed during the high neutron fluence, which would hinder dislocations slip and lead to irradiation hardening; Second, a large density of dislocation loops would be formed during the high neutron fluence, and the dislocation loops could also hinder the dislocations slip, resulted in irradiation hardening; Third, a small number of defects were formed during the low neutron fluence, and these small defects were invisible dislocation loops, which were also the initial source of radiation hardening. Ma et al. [67] observed the change of microstructure under different irradiation damage (0.08 dpa and 1.5 dpa) by TEM and studied the relationship between irradiation hardness and irradiation damage. They found that the number density and size of black dots increased significantly, dislocation entanglement formed dislocation networks, and high-density dislocation defects increased significantly with the increase of radiation damage from 0.08 dpa to 1.5 dpa, which would lead to the increase of SA508Gr.3 steel hardness from 3.0 GPa to 4.2 Gpa.

To investigate the effect of irradiation on mechanical properties, Liu et al. [71] established a crystal plastic constitutive model based on the irradiation dislocations density and irradiation defects, and combined the experimental methods to study the effect of irradiation on the tensile strength and irradiation embrittlement of SA508Gr.3 steel. They found that the irradiation defects could effectively hinder dislocations motion, resulting in higher strength and lower toughness of the material, and the experimental results were in good agreement with the finite element simulation results. Marini et al. [72] studied the evolution law of mechanical properties of SA508Gr.3 steel with three different microstructures after irradiation. The results showed that the UTS of bainite SA508Gr.3 increased by 12%, bainite-martensite increased by 13% and martensite increased by 12%. The variation law of UTS was shown in Table 8. In addition, the ductile–brittle transition temperature (DBTT) of bainite SA508Gr.3 increased by 50 °C, that of bainite–martensite increased by 75 °C and that of martensite increased by 57 °C.

To characterize the effect of irradiation on embrittlement, Zhou et al. [73] compared the impact properties of SA508Gr.3 steel before and after irradiation. They found that irradiation decreased the dimple area of the impact fracture and increased the area of the radiation zone. The upper shelf energy decreased by 61 J, and the ductile–brittle transition temperature increased by 68 °C and the irradiated specimens exhibited significant radiation embrittlement, which was mainly attributed to the decrease of the crack initiation and propagation energy after irradiation. Lee et al. [74] studied the irradiation hardening and irradiation embrittlement behavior of SA508Gr.4N steel with different Ni contents (0.9–3.5 wt.%) under different irradiation doses. The investigation found that the difference of Ni content had little effect on the irradiation hardening and embrittlement of neutron irradiated SA508Gr.4N steel, while the increase of hardness and ductile–brittle transition temperature (DBTT) after neutron irradiation was mainly affected by the irradiation doses. The hardness and DBTT of SA508Gr.4N steel increased with the increase of irradiation doses. Slugen et al. [68] studied the mechanism of irradiation embrittlement of 15Kh2MFAA steel for nuclear pressure vessels by using positron annihilation technology. They found that neutron irradiation resulted in the formation of small cluster structures, which would prevent the free movement of dislocations, thus leading to the increase of ductile and brittle transition temperature of materials, resulting in irradiation embrittlement. Mamivand et al. [75] developed an improved Cu-rich and Mn–Ni–Si-phase co-precipitation Cluster Dynamics model and verified the accuracy of the model through experiments. They found that the alloying elements Cu and Ni were the main elements leading to embrittlement of nuclear pressure vessel materials through model and experiments. The Cu precipitated rapidly under irradiation conditions, which significantly reduced the irradiation fluence marking the beginning of embrittlement; Cu element was the main factor of irradiation embrittlement when Cu content was less than 0.1 at.%; Cu-rich precipitates were the main nucleation site of RPV materials embrittlement when Cu content was greater than 0.1 at.%. In addition, the in-cascade nucleation was the main nucleation mechanism of irradiation embrittlement when Ni content was low and medium. The homogeneous nucleation was the main nucleation mechanism of irradiation embrittlement when Ni content was greater than 1.5%. Moreover, Loat et al. [76] studied the effect of 450 °C annealing for 40 h on irradiation embrittlement of SA508Gr.2 steel for nuclear pressure vessels. In low-Cu RPV steel, Ni–Mn–Si-rich clusters would hinder the movement of dislocations, which was one of the main reasons for radiation embrittlement. The mechanical properties of the tested specimens were significantly recovered when annealed at 450 °C, which was mainly due to the dissolution of Ni–Mn–Si-rich clusters and the recovery of matrix defects.

In conclusion, nuclear pressure vessel materials with different microstructures have different irradiation properties. Irradiation can cause microstructure matrix defects, irradiation hardening and irradiation embrittlement, and the degree of irradiation hardening and embrittlement increases with the increase of irradiation doses. Generally, the matrix damage, Cu-rich cluster structures, Ni–Mn–Si-rich clusters as well as P, S segregation on grain boundaries were important factor for radiation embrittlement of nuclear pressure vessel materials. However, the clusters would decompose and the defects would recover during the annealing process, which was beneficial to improve the properties of the materials. 

## 5. Corrosion Properties of Steels for Nuclear Pressure Vessel

Theoretically, nuclear pressure vessel materials are rarely in direct contact with corrosive solutions due to the austenitic stainless surfacing on the inner wall of nuclear pressure vessels. However, the actual operation experience of global nuclear power plants shows that the serious corrosion behavior of RPV materials caused by the leakage of boric acid water in the primary circuit is common [27,77]. Therefore, the research on the corrosion resistance of nuclear pressure vessels needs to be given more attention in order to ensure the safe service.

According to the operating experience of PWR nuclear power plants in the world, the corrosion behavior of nuclear pressure vessel materials caused by boric acid leakage could be divided into the following two types [27,78,79]: First, the overlaying layer of stainless steel might be damaged when the materials for RPV had been used for a long time in harsh service environment, which led to the RPV materials directly contacting with boric acid water in the primary circuit, and then resulted in corrosion behavior; Second, boric acid water leaked out from the system, such as flanged joints, sealing gaskets, bolts and alloy pipe penetrations components, and contacted with high temperature low-alloy steel components, which resulted in the increase of boric acid solution concentration as well as the decrease of pH, and then accelerated the corrosion of RPV materials.

**Figure 3:**
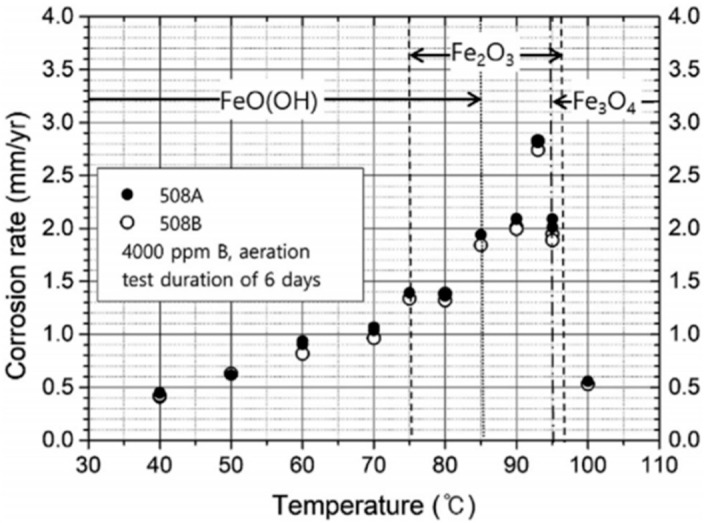
Corrosion rates and corrosion products of 508A and 508B in boron concentration of 4000 ppm [81].

In the service environment of nuclear pressure vessel, the boric acid concentration and temperature were the main factors affecting the compactness of passivation film of low-alloy steel. Xiao et al. [27] found that the corrosion rate of SA508Gr.3 increased with the augmenting of boric acid concentration and temperature in deaerated solutions. Xia et al. [80] investigated the influence of temperature on the corrosion behavior of SA508Gr.4N steel by using electrochemical noise technology, and found that the noise resistance of specimen decreased with the increase of temperature from 27 °C to 250 °C, indicated that the increase of corrosion rate. The corrosion resistance of material was closely related to the passive film. The passive film formed on the surface of the material was easier to degrade during the environment of high temperature and pressure corrosion, which due to the characteristic of porous structure [41]. Lim et al. [81] thought that the temperature had a complex effect on the corrosion rate of materials. They tested the effects of boric acid concentration, immersion time and temperature on the corrosion rate of SA508Gr.3 steel in boric acid solution by weight loss method and found that the corrosion rate of specimen increased with the augment of boric acid concentration and decreased with the augment of immersion time. However, the corrosion rate of SA508Gr.3 steel in boron concentration of 4000 ppm first increased and then decreased with the increase of temperature (Figure 3), which due to the concentration of dissolved oxygen in water would decrease rapidly with the increase of temperature. Therefore, the corrosion products were conducive to the formation of FeO(OH) and Fe_2_O_3_ at low temperature, while the corrosion products were favorable to the formation of Fe_3_O_4_ at high temperature. The adhesive strength of latter with interface was higher than that of the former, which protected the material and decreased the corrosion rate to very low values even at high temperature.

At present, it can be determined that the corrosion rate of nuclear pressure vessel materials increases with the increase of boric acid concentration, but there are different views on the influence of temperature on its corrosion rate. In addition, the research reports on the corrosion resistance of nuclear pressure vessel materials are not systematic. The structure of passive film, the formation and destruction mechanism of passive film, and the anti-corrosion measures are not clear. Therefore, in order to ensure the long-term safe service of nuclear pressure vessels, the research on corrosion resistance of nuclear pressure vessels materials needs to be focused on in future research work.

## 6. Study on Thermal Aging of Steel for Nuclear Pressure Vessel

Thermal aging refers to a phenomenon that the microstructure of material will change under high temperature environment for a long time, and then lead to changes in the properties. Long term service of RPV materials in high temperature environment can easily cause thermal aging embrittlement.

The thermal aging embrittlement of low-alloy steel is closely related to thermal aging time and temperature. Druce et al. [82] studied the thermal aging behavior of RPV materials at 300–550 °C for 20,000 h, and the results showed that the ductile-brittle transition temperature increased with the increase of temperature and aging time, which was mainly attributed to phosphorus segregation and carbides coarsening at grain boundaries. Wang et al. [83] studied the changes of microstructure and mechanical properties of low-activity martensitic steel for reactor under thermal aging 550 °C and different holdings time. The results showed that the grain size of the material increased, and the width of martensitic lath widens with the elapsed of thermal aging time to 10,000 h. In addition, the thermal aging had little effect on the tensile strength of low-activity martensitic steel, but the ductile–brittle transition temperature increased by 46 °C. Xing et al. [38] investigated the effect of thermal aging of 16MnD5 steel at 500 °C for 0 h, 1000 h, 3000 h and 5000 h on mechanical properties and found that thermal aging could decrease the impact toughness and increase the brittleness of materials, and the ductile–brittle transition temperature of materials increases significantly with the extension of thermal aging time. Meanwhile, the type of impact fracture was transformed from ductile fracture to brittle fracture. Shtrombakh et al. [35] compared the mechanical properties of 15Kh2NMFA-A base material and Sv-10KhGNMAA weld material for VVER-1000 nuclear pressure vessel for thermal aging 200,000 h. They found that with the increase of thermal aging time, the grain boundary segregation of weld metal became more serious, the critical brittleness temperature increased and the proportion of brittle intergranular fracture increased monotonically, while the base metal did not change significantly, which was mainly due to the formation of impurity segregation at the grain boundary of weld metal.

It can be seen that the long-term thermal aging may not have a significant influence on mechanical properties but has a great influence on the ductile–brittle transition temperature. Generally, the ductile–brittle transition temperature of RPV materials increases significantly with the increase of thermal aging time.

## 7. Fatigue Properties of Steels for Nuclear Pressure Vessel

Fatigue damage accompanied the whole service cycle of RPV, and it mainly includes two influencing factors: Material factors and environmental factors, also known as internal and external factors [84,85,86,87,88,89,90]. The essential characteristic of materials was that internal factors affected the fatigue properties, which had a decisive effect on fatigue crack initiation, cyclic hardening/softening and fatigue life. The results of some literature showed that the chemical composition, microstructures and inclusions have great influence on the fatigue properties of materials [42,56,85,91,92]. Environmental factors were the external factor that affected the fatigue properties. The influencing factor of environment on the fatigue properties of RPVs materials mainly included service environment, loading environment and natural environment, among which service environment and loading environment were common influencing factors [87,93,94,95,96,97]. Service environment included service temperature, pressure, water environment, dissolved hydrogen/oxygen and pH value, and so on. Loading environment included loading frequency, loading wave, stress ratio, stress amplitude, strain amplitude as well as the strain rate, and so on.

### 7.1. Internal Factors

The influence of microstructures structure, inclusions, carbides and HAGBs on the fatigue properties of nuclear pressure vessel materials are internal factors. Jang et al. [84] studied the fatigue crack initiation and propagation behavior of pearlite SA508Gr.1 steel and bainite SA508Gr.1 steel. They found that the former would have a larger local stress concentration at the ferrite–pearlite phase boundary during the fatigue deformation, which would cause crack initiation and faster crack propagation, and then decrease the fatigue life of pearlitic SA508Gr.1 steel. On the contrary, the fine carbides in bainite SA508Gr.1 steel could hinder the crack propagation, which could increase the fatigue life of SA508Gr.1 steel. Atkinson et al. [98] studied the effect of different sulfur contents on the corrosion fatigue properties of A533B steel under high temperature and high pressure environment, and found that the corrosion fatigue crack propagation rate increased significantly with the increase of sulfur contents. The change of sulfur contents changed the electrode potential of A533B steel in water environment, which would accelerate the corrosion fatigue behavior of specimen, and then increase the fatigue crack propagation. In addition, sulfur was also a harmful element in alloy steel, which could easily cause segregation and deteriorate the fatigue resistance of material. Singh et al. [85] studied the influence of prior austenite grain boundaries on crack propagation, and found that the crack propagation was hindered and the crack propagation rate was decreased when the fatigue crack tip encountered the prior austenite grain boundaries. Prior austenite grain boundaries belonged to HAGBs. Some scholars [19,99,100] believed that the HAGBs could hinder crack propagation. Compared with low-angle grain boundaries (LAGBs), the HAGBs had higher grain boundary energies owing to the atoms were more active and deviated from their equilibrium positions [19,100,101]. The irregular arrangement of atoms would consume more crack propagation energy when it encountered the HAGBs, and the crack with low propagation energy was easier to be arrested by the HAGBs. Therefore, the HAGBs could effectively hinder the crack propagation and decrease the crack propagation rate.

**Figure 4:**
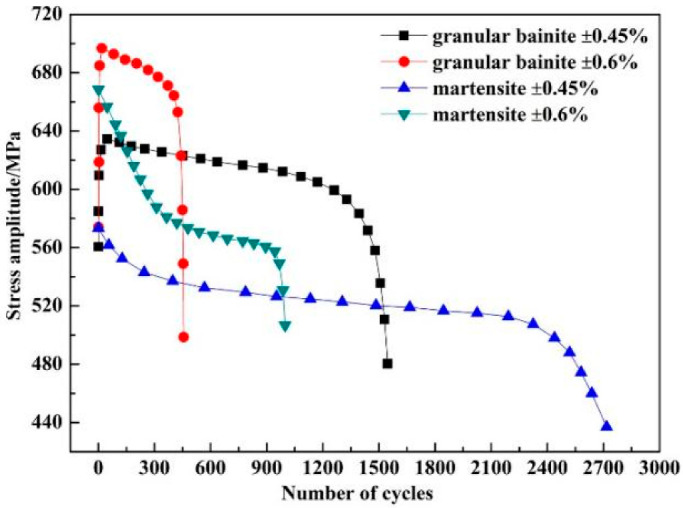
Fatigue life cycles curve of specimens with granular bainite and martensite at different strain amplitudes [40].

The new generation of RPV pay more attention to safety and service life compared with the previous RPVs materials, so the forging size and wall thickness of SA508Gr.4N steel were greatly increased [19,102]. The increase of wall thickness would cause the difference in the cooling rate along the wall thickness direction during the quenching and cooling, resulted in the formation of martensite SA508Gr.4N steel on and near the surface due to the higher cooling rate, and the formation of granular bainite SA508Gr.4N steel in the core due to the lower cooling rate. Dai et al. [40] then compared the fatigue properties of martensite SA508Gr.4N steel and granular bainite SA508Gr.4N steel, and found that the former had better fatigue properties under the same strain amplitude (Figure 4), which was mainly attributed to the former having fewer crack initiation points, narrower fatigue striations separation and more HAGBs volume fraction. Fewer crack initiation points meant less fatigue cracks, narrower fatigue striations separation meant slower crack propagation rate and more HAGBs volume fraction could more effectively hinder fatigue crack propagation. These reasons led to the higher fatigue life of martensite SA508Gr.4N steel. Therefore, the granular bainite SA508Gr.4N steel was the weak link of RPV suffering fatigue damage during the service conditions. The study on the weak link of fatigue damage of RPV was beneficial to improve its service cycle.

**Figure 5:**
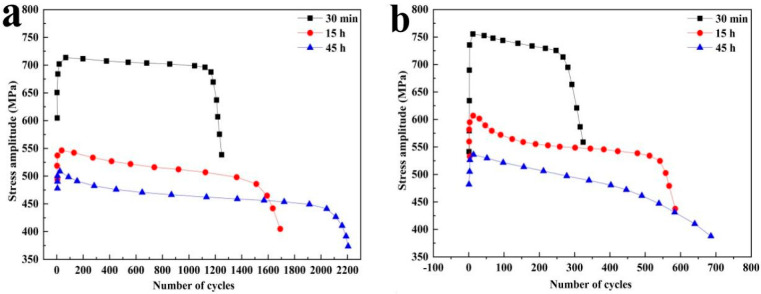
The fatigue life cycles curve of granular bainite during different tempering durations: (**a**) strain amplitude ±0.45% and (**b**) strain amplitude ±0.6% [56].

They [56] followed studied the influence of microstructure evolution on granular bainite SA508Gr.4N steel low cycle fatigue properties, and found that the fatigue life increased monotonously with the increase of tempering time (Figure 5). More M/A islands decomposed into fine carbides with the increase of tempering time. The M/A islands decomposition could effectively release the local stress concentration of the material, resulting in the reduction of fatigue initiation points during the fatigue deformation. In addition, the fine carbides derived from the decomposition of M/A islands could also hinder fatigue crack propagation and decrease the crack propagation rate. Therefore, the fatigue life of granular bainite SA508Gr.4N steel would increase with the increase of tempering time.

### 7.2. External Factors

The loading waveform of fatigue test can be divided into triangle waveform, sinusoidal waveform and positive sawtooth waveform, and so on. The difference of waveform will lead to the difference of fatigue properties. Achilles et al. [87] studied the effect of sinusoidal waveform and positive sawtooth waveform on fatigue crack propagation, and found that the crack propagation rate of positive sawtooth wave was always faster than that of sinusoidal waveform during the fatigue test. This might be because the loading time of the positive sawtooth waveform was longer than that of the sinusoidal waveform. The longer the load time, the more hydrogen was transported to the maximum axial stress region in the environment, resulted in the faster crack propagation rate.

Huang et al. [94] investigated the effects of air and water environment on fatigue crack propagation rate of SA533B steel for RPV, respectively, and the results showed that the fatigue crack propagation rate in water environment was higher than that in air. Subsequently, they [95] studied the effect of temperature on the fatigue crack propagation rate, and found that the fatigue crack propagation rate increased with the increase of temperature. Shi et al. [103] studied the effect of strain amplitude on fatigue life of SA508Gr.3 steel and found that the fatigue life decreased with the increase of strain amplitude. Fatigue damage was the result of plastic strain accumulation. The greater the fatigue strain, the greater the degree of plastic deformation of the material, and the greater the fatigue damage accumulation, resulting in the lower fatigue life of the materials.

**Figure 6:**
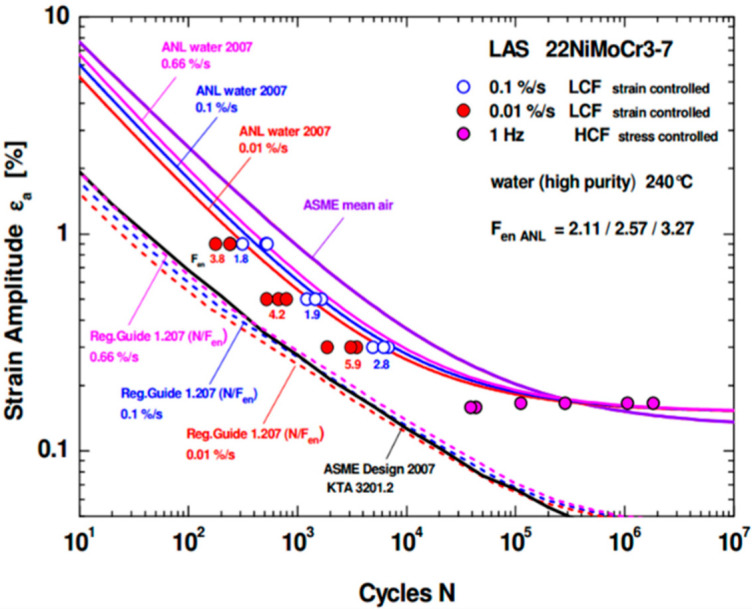
Effect of strain rates on fatigue life of 22NiMoCr3-7 steel [97].

In addition, the loading frequency and strain rate also had great influence on the fatigue properties of nuclear pressure vessel materials. Tice [96] investigated found that the higher the loading frequency, the lower the fatigue life of materials. Herter et al. [97] studied the effect of strain rate on the fatigue life of 22NiMoCr3-7 steel for RPV, and found that the fatigue life increased with the decrease of strain rate under the same conditions, as shown in Figure 6.

The effects of carbides, HAGBs and microstructure evolution on fatigue properties are internal factors. Fine carbides and HAGBs can hinder fatigue crack propagation and increase the fatigue life of materials. The effects of strain amplitude, service environment and loading frequency on fatigue performance are external factors. The increase of strain amplitude and corrosive environment will decrease the fatigue life of materials.

## 8. Conclusions and Outlook

In this review, we have summarized the research progress of nuclear pressure vessels from different perspectives, including the development of RPV materials, service environment, microstructure evolution, mechanical properties after forging, as well as the effect of service environment on RPV materials. Based on the improvement of nuclear power safety and economy, nuclear pressure vessels had developed from the early plate welding structure to the present large-scale integrated forging structure. The low-temperature and high-temperature forging process were not conducive to the uniformity of the microstructure of the material after forging. Therefore, the selection of forging parameters was very important for the subsequent materials properties before the application of nuclear pressure vessels. The ASTM standard specified that nuclear pressure vessels must meet certain performance requirements after forging. The alloying elements, heat treatment parameters determined the mechanical properties of materials by changing the microstructure structure, grain size, carbides size and large angle grain boundary volume fraction.

In addition, nuclear pressure vessels had been used in harsh environments such as neutron irradiation, corrosion, high temperature thermal aging and fatigue damage for a long time, which would deteriorate the properties of RPV materials. Nuclear pressure vessel materials exposed to neutron irradiation for a long time would cause matrix damage, dislocation loops and impurity element segregation, resulted in irradiation hardening and irradiation embrittlement. Although stainless steel was overlaid on the inner wall of the nuclear pressure vessel to prevent corrosion of its materials, long-term service might lead to damage of stainless steel and leakage of potential leakage sources, which would lead to directly contact between the RPV materials and boric acid corrosion solution, and then cause the occurrence of corrosion behavior. Long-term service at high temperature would cause thermal aging behavior of RPV materials, which would lead to microstructure decomposition or carbides coarsening. The thermal aging behavior mainly caused the increase of the ductile–brittle transition temperature and deteriorated the impact properties of materials. Finally, fatigue damage also accompanied the whole service process of nuclear pressure vessels. The influence factors of fatigue included microstructure evolution, second phase, service environment, corrosion environment and strain rate, etc. The fine second phase could hinder the propagation of fatigue cracks, while the coarsening second phase would become the source of crack initiation. In addition, corrosion fatigue would significantly decrease the fatigue life of materials compared with fatigue in air.

At present, the research on the nuclear pressure vessel is mostly the influence of a single factor, such as radiation, corrosion and fatigue. However, the service environment of nuclear pressure vessels is very complex, and the influence of single factor on the performance is far from the real service conditions, so the research results are insufficient and unscientific for the safety application of RPV materials. Therefore, in order to ensure their safe service in the later period of service, the collaborative mechanism of multiple service environments on nuclear pressure vessel materials should be focused on studied in future work.

## Figures and Tables

**Table 1:** The content of main alloying elements of reactor pressure vessel steel for PWR (wt.%) [24,26].

Materials	C	Si	Mn	Cr	Ni	Mo
A212B	≤0.30	0.15–0.30	0.85–1.20	-	-	-
A302B	≤0.26	0.13–0.32	1.10–1.55	-	-	0.41–0.64
A533B	≤0.25	0.15–0.30	1.51–1.50	-	0.40–0.70	0.45–0.60
A508-2	≤0.27	0.15–0.35	0.50–0.90	0.25–0.45	0.50–0.90	0.55–0.70
US A508-3	≤0.26	0.15–0.40	1.20–1.50	≤0.25	0.40–1.00	0.45–0.55
20MnMoNi55	0.17–0.23	0.15–0.30	1.20–1.50	≤0.20	0.50–1.00	0.40–0.55
22NiMoCr37	≤0.20	0.15–0.30	1.20–1.40	≤0.40	0.40–1.00	0.40–0.55
16MND5	≤0.20	0.10–0.30	1.15–1.55	≤0.25	0.50–0.80	0.45–0.55
SFVV3	0.15–0.22	0.15–0.35	1.40–1.50	0.06–0.20	0.70–1.00	0.46–0.64
Chinese A508-3	0.19	0.19–0.27	1.20–1.43	0.06–0.12	0.73–0.79	0.48–0.51
15X2HMΦA	0.13–0.18	0.17–0.37	0.30–0.60	1.80–2.30	1.00–1.50	0.50–0.70
A508-4	≤0.23	≤0.40	0.20–0.40	1.50–2.0	2.80–3.90	0.40–0.60

**Table 2:** The chemical composition of A508 series steel (wt.%) [24].

Elements	Grade 1	Grade 2	Grade 3	Grade 4N	Grade 5	Grade 6
C (max)	0.35	0.27	0.25	0.23	0.23	0.28–0.33
Si (max)	0.40	0.40	0.40	0.40	0.30	0.35
Mn	0.40–1.05	0.50–1.00	1.20–1.50	0.20–0.40	0.20–0.40	0.75–1.15
Cr	≤0.25	0.25–0.45	≤0.25	1.50–2.00	1.50–2.00	0.70–1.00
Ni	≤0.40	0.50–1.00	0.40–1.00	2.80–3.90	2.80–3.90	0.75–0.95
Mo	≤0.10	0.55–0.70	0.45–0.60	0.40–0.60	0.40–0.60	0.30–0.45

**Table 3:** Neutron fluence rate and neutron fluence in common reactor (E > 1 MeV) [31].

Reactor Type	Flux, m^−2^·s^−1^ (E > 1 MeV)	Lifetime * Fluence, m^−2^ (E > 1 MeV)
WWER-440 core weld	1.2 × 10^15^	1.1 × 10^24^
WWER-440 maximum	1.5 × 10^15^	1.6 × 10^24^
WWER-1000	3−4 × 10^14^	3.7 × 10^23^
PWR (W)	4 × 10^14^	4 × 10^23^
PWR (B&W)	1.2 × 10^14^	1.2 × 10^23^
BWR	4 × 10^13^	4 × 10^22^

* Lifetime fluences for WWERs are calculated for 40 calendar years, PWRs are calculated for 32 Effective Full Power Years. However, note that this does not include the effect of service or operational life extension.

**Table 4:** The mechanical properties requirements [24].

Mechanical Properties	Grades 1 and 1a	Grades 2 Class 1 and 3 Class 1	Grades 2 Class 2 and 3 Class 2	Grades 4N Class 1 and 5 Class 1	Grades 4N Class 2 and 5 Class 2	Grades 6 Class 1	Grades 6 Class 2
Tensile strength, ksi [MPa]	70–95 [485–655]	80–105 [550–725]	90–115 [620–795]	105–130 [725–895]	115–140 [795–965]	85–110 [585–760]	95–120 [655–825]
Yield strength, min [0.2% offset], ksi [MPa]	36 [250]	50 [345]	65 [450]	85 [585]	100 [690]	60 [415]	75 [515]
Elongation in 2 in. or 50 mm, min, %	20	18	16	18	16	20	18
Reduction of area, min, %	38	38	35	45	45	35	35
Minimum average value of set of three specimens, ft·lbf [J]	15 [20] (4.4 °C)	30 [41] (4.4 °C)	35 [48] (21 °C)	35 [48] (−29 °C)		20 [27] (−59 °C)	
Minimum value of one specimen, ft lbf [J]	10 [14] (4.4 °C)	25 [34] (4.4 °C)	30 [41] (21 °C)	30 [41] (−29 °C)		15 [20] (−59 °C)	

**Table 5:** Chemical composition of steel (wt.%) [51].

	C	Ni	Cr	Mo	Fe
KL4-Ref.	0.21	3.59	1.79	0.54	Bal.
KL4-Ni 1	0.22	2.66	1.81	0.53	Bal.
KL4-Ni 2	0.21	4.82	1.83	0.54	Bal.
KL4-Cr 1	0.21	3.65	1.04	0.54	Bal.
KL4-Cr 2	0.21	3.63	2.47	0.53	Bal.
KL4-Mo 1	0.21	3.57	1.87	0.11	Bal.
KL4-Mo 2	0.21	3.7	1.86	1.02	Bal.

**Table 6:** Tensile and Charpy test results of the test steel [51].

	KL4-Ref.	KL4-Ni 1	KL4-Ni 2	KL4-Cr 1	KL4-Cr 2	KL4-Mo 1	KL4-Mo 2
YS (MPa)	581	535	677	585	590	533	633
UTS (MPa)	750	698	820	762	762	735	808
USE (J)	226	262	207	189	216	231	184
T28J (°C)	−140	−94	−176	−77	−149	−146	−126
T41J (°C)	−128	−87	−161	−65	−138	−136	−114

**Table 7:** Fraction of tested materials with different cooling rates and tensile properties [54].

Cool Rate (°C/min)	Martensite (%)	Bainite (%)	Austenite (%)	Yield Strength (MPa)	Tensile Strength (MPa)	USE (J)
3	0	94	6	531	740	200
28.2	69	13	18	545	759	224
960	99	0	1	573	742	269

**Table 8:** The change of UTS and DBTT of different microstructures before and after irradiation (MPa/°C) [72].

Irradiated	Bainite	Bainite–Martensite	Martensite
Un-irradiated	638/−54	698/−77	751/−116
Irradiated	717/−4	786/−2	838/−59
Increase/shift	12/50	13/75	12/57

## Data Availability

Not applicable.
